# Supplementary material for: Coastal El Niño triggers rapid marine silicate alteration on the seafloor
Source: Nat Commun. 2023 Mar 25;14:1676. doi: 10.1038/s41467-023-37186-5 (PMC10039921; doi:10.1038/s41467-023-37186-5)
Supplement: Supplementary file 1 — Supplementary Information [file 41467_2023_37186_MOESM1_ESM.pdf]

Supplement to

Coastal El Niño triggers rapid marine silicate alteration on the seafloor

Sonja Geilert[1*], Daniel A. Frick[2], Dieter Garbe-Schönberg[3,4], Florian Scholz[1], Stefan Sommer[1], Patricia Grasse[1,5], Christoph Vogt[6], Andrew W. Dale[1]

[1]GEOMAR Helmholtz Centre for Ocean Research Kiel, 24148 Kiel, Germany
[2]GFZ German Research Centre for Geosciences, Section Earth Surface Geochemistry, Telegrafenberg, 14473 Potsdam, Germany
[3]Institute of Geosciences, University of Kiel, 24118 Kiel, Germany
[4]Department of Physics and Earth Sciences, Jacobs University Bremen, 28759 Bremen, Germany
[5]German Centre for Integrative Biodiversity Research (iDiv) Halle-Jena-Leipzig, 04103 Leipzig, Germany
[6]Faculty of Geosciences/Crystallography and Geomaterials & MARUM, University of Bremen, 28359 Bremen, Germany

*Corresponding author: sgeilert@geomar.de

1. Supplementary figures to main text

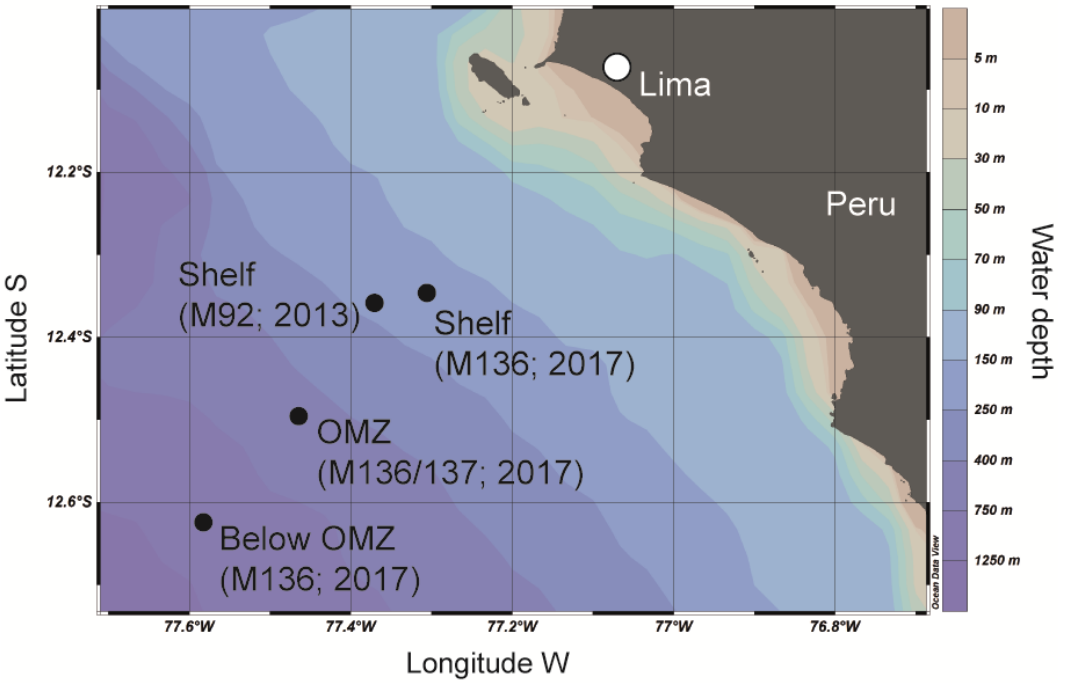

Figure S1: Sampling location and stations on the Peruvian margin in 2013 (cruise M92) and in 2017 (cruise M136/137). At all stations, sediments were sampled for pore fluids and solids, and in situ benthic chamber incubations were conducted.

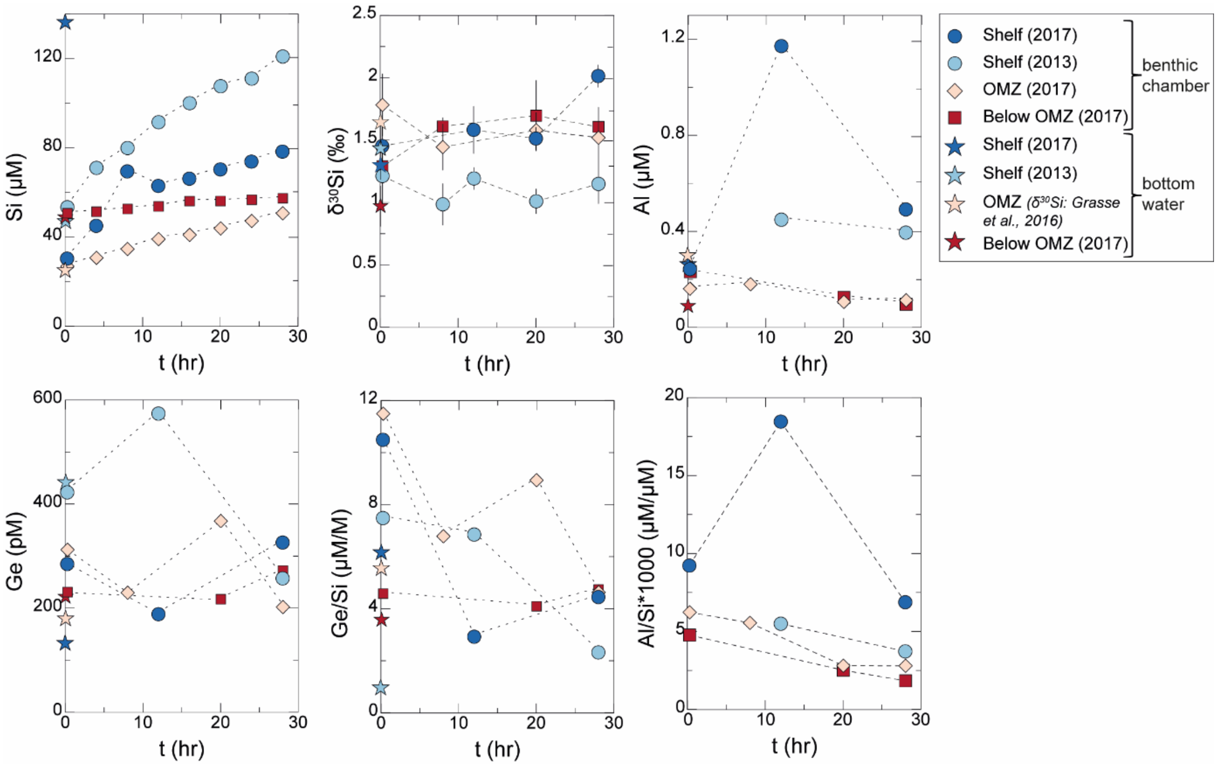

Figure S2: In situ benthic chamber incubation experiments off Peru. Geochemical parameters and Si isotope (δ^{30}Si) values are shown versus incubation time (hours; hr). Bottom water values are also shown (Oxygen Minimum Zone (OMZ) bottom water δ^{30}Si data are from Grasse et al.[1]).

2. Supplementary tables to main text

Table S1: Research cruise designation, station/gear, water depths and year of the sampling campaigns off the Peruvian coast. Coordinates of the sampling stations are given in Krahmann et al.[2]

Cruise	Station	Station/ gear	water depth (m)	Sampling year
M136	Shelf	483/MUC8	74	2017
		533/BIGO2-4	74	
M92	Shelf	054/ MUC13	71	2013
		110/BIGO1-2	74	
M136	OMZ	574/MUC10	302	2017
M137		791/BIGO1-5	300	
M136	Below OMZ	543/MUC9	750	2017
		460/BIGO2-2	752	

Table S2: Pore fluid and solid geochemical analyses and pore fluid Si isotope analyses. cmbsf = centimeters below sea floor, bw = bottom water.

				Pore fluids						Solids	
Depth	Si	Al	Ge	Ge/Si	Al/Si*10^3	δ^{30}Si	2SD	δ^{29}Si	2SD	Al	K
(cmbsf)	(µM)	(µM)	(pM)	(µM M^{-1})	(µM $µM^{-1}$)	(‰)	(‰)	(‰)	(‰)	mg g^{-1}	mg g^{-1}
M136/MUC8; Shelf 2017; 74m water depth											
bw	145	0.23	138	5.06	8.54	1.30	0.11	0.73	0.13		
0.5	429	0.71	1225	2.87	1.67	3.72	0.14	1.84	0.15	50.6	16.2
1.5	678	1.00	1349	2.36	1.75	3.12	0.15	1.64	0.07	53.0	16.4
2.5	838									54.5	16.6
3.5	916									51.3	16.1
4.5	980	0.79	1996	2.28	0.90	1.94	0.15	1.01	0.05	50.3	16.0
5.5	1005	0.64	2258	2.49	0.70	1.98	0.22	1.02	0.11	47.2	15.5
7	1019									49.0	15.7
9	971									57.1	17.0
11	961									57.1	16.9
13	955	0.74	2148	2.45	0.85	1.35	0.14	0.69	0.18	57.4	17.4
15	942									63.5	19.2
20	912									55.8	16.7
24	907	0.86	1239	1.50	1.04	1.26	0.07	0.55	0.04	58.2	17.2
28	930									56.7	17.0
32.5	928	0.94	1060	1.30	1.16	1.15	0.16	0.59	0.05	59.6	17.5
M92/MUC13; Shelf 2013; 71m water depth											
bw	44		441	6.54		1.43	0.18	0.70	0.14		
0.25	653										
0.75	584										
1.25	786	0.19	730	1.01	0.26	1.33	0.14	0.69	0.10	41.4	13.4
1.75	688										
2.25	800										
2.75	832										
3.25	867									52.9	15.5
3.75	878										
4.5	888										
5.5	882	1.01	1349	1.62	1.22	1.24	0.07	0.64	0.19	57.6	16.2
6.5	885										
7.5	868										
8.5	873									53.1	15.2
9.5	871										
11	890										
13	883										
15	877										
17	846										
19	898										
23	912										
27	938										

31	921										
35	904										
39	905	0.74	1294	1.57	0.90	1.09	0.10	0.55	0.10	61.1	16.6
43	891										
M136/MUC10; OMZ 2017; 302 m water depth											
bw	31	0.34	179	5.54	10.39						
0.5	158	0.39	206	1.34	2.53	1.19	0.09	0.54	0.19	22.1	10.2
1.5	236									18.7	9.0
2.5	311									27.4	11.1
3.5	357									29.0	11.2
4.5	394									30.1	10.9
5.5	429									33.0	11.5
7	457									31.1	11.7
9	473	0.41	509	1.10	0.89	1.34	0.11	0.74	0.10	31.2	11.4
11	488									35.3	11.8
13	498									32.2	11.0
15	534										
20	552									21.7	9.6
24	560									21.6	9.8
28	568					1.23	0.14	0.62	0.07	31.9	11.8
M136/MUC9; Below OMZ 2017; 750 m water depth											
bw	59	0.09	220	3.79	1.60	1.04	0.16	0.53	0.18		
0.5	125	0.40	234	1.91	3.26	1.07	0.19	0.46	0.08	28.7	36.6
1.5	164									29.9	35.6
2.5	170									31.9	33.9
3.5	171									31.2	35.9
4.5	170									33.0	34.0
5.5	169		358	2.19		0.53	0.05	0.28	0.06	34.6	33.3
7	145									34.6	34.7
9	140									29.6	37.1
11	165									31.9	38.8
13	163									32.3	36.5
15	173									37.5	35.4
20	190	0.83	330	1.81	4.56	0.75	0.14	0.39	0.10	37.9	34.2
24	206									36.7	34.0

Table S3: XRD results for shelf stations from 2017 (M136/MUC8) and 2013 (M92/MUC13). Note that only main minerals are reported and that halite precipitated during freeze-drying (between 10 wt% in 2017 at 32.5 cmbsf and 70 wt% in 2013 at 0.75cmbsf; average of all stations = 21 wt%).

Station	Depth (cmbsf)	Qz (wt%)	Ab (wt%)	An,Or,Mc (wt%)	Ms (wt%)	Clc (wt%)	Ilt (wt%)	Amp (wt%)	Pyr (wt%)	Cal (wt%)
Shelf 2017 (M136/ MUC8)	0.5	29	19	6 (Mc)	11	5	/	5	2	2
	2.5	20	25	/	19	5	/	5	1	3
	4.5	23	27	/	13	/	/	/	3	3
	5.5	22	15	11 (Mc)	/	/	/	7	2	2
	13	23	17	11 (Mc)	17	7	/	6	2	/
	32.5	12	31	18 (Or)	21	/	/	/	3	/
Shelf 2013 (M92/ MUC13)	0.75	9	/	13 (An)	/	/	/	/	/	3
	1.25	15	22	10 (Mc)	15	5	/	/	/	/
	1.75	16	18	/	26	3	14	4	1	/
	3.25	19	27	8 (Mc)	24	/	/	/	4	/
	5.5	20	21	/	/	5	22	/	2	/
	39	33	19	/	10	6	/	7	3	3

Qz=quartz, Ab=albite, An=anorthite, Or=orthoclase, Mc=microcline, Ms=muscovite, Clc=clinochlore, Ilt=illite, Am=amphibole, Pyr=pyrite, cal=calcite, Gp=gypsum

Table S4: In situ Si isotope and Al/Si ratios based on intensity signals. Mass ratios of Al and Si were calculated based on the GeoReM[3] database using the measurements of the USGS reference material BHVO-2G as calibration standard.

Sample-ID	Type	$\delta^{30}Si$ (‰)	2SE (‰)	$\delta^{29}Si$ (‰)	2SE (‰)	$^{27}Al/^{28}Si$	2SE*10^{2a}	Al/Si (wt% wt%$^{-1}$)
M136-1	Authigenic clay	-0.46	0.10	-0.25	0.08	0.71	0.49	0.38
M136-2		0.33	0.14	0.17	0.14	0.17	1.50	0.08
M136-3		-0.23	0.12	-0.14	0.09	0.51	1.28	0.27
M136-4		-0.16	0.11	-0.10	0.09	0.41	0.50	0.22
M136-5		0.12	0.16	0.15	0.17	0.48	1.08	0.26
M136-6		-0.58	0.13	-0.29	0.09	0.75	0.68	0.40
M136-7		0.11	0.13	0.01	0.10	0.31	0.40	0.16
M136-M1	Matrix	0.11	0.12	0.08	0.09	0.40	0.39	0.21
M136-M2		-0.01	0.11	0.03	0.09	0.41	0.49	0.22
M136-M3		0.08	0.10	-0.08	0.09	0.41	0.36	0.22
M136-M4		-0.04	0.08	0.03	0.07	0.41	0.43	0.22
M136-M5		0.02	0.07	0.03	0.07	0.40	0.43	0.21
M136-M6		-0.01	0.09	0.05	0.08	0.41	0.33	0.22
M136-M7		0.07	0.10	0.02	0.07	0.40	0.55	0.21
M92-1a	Matrix	0.25	0.09	0.11	0.06	0.16	0.61	0.08
M92-1b		0.31	0.09	0.16	0.06	0.16	0.61	0.08
M92-2a		0.39	0.09	0.11	0.07	0.16	0.58	0.08
M92-2b		0.25	0.08	0.19	0.06	0.19	0.66	0.09
M92-3kl		0.19	0.09	0.11	0.06	0.16	0.67	0.08

M92-3a	0.21	0.08	0.16	0.06	0.15	0.71	0.08
M92-3b	0.15	0.08	0.14	0.07	0.14	0.72	0.07
M92-4a	0.28	0.09	0.11	0.06	0.15	0.70	0.07
M92-4b	0.23	0.09	0.15	0.07	0.16	0.75	0.08
M92-5a	0.24	0.09	0.06	0.07	0.15	0.70	0.08
M92-5b	0.28	0.08	0.13	0.06	0.14	0.67	0.07
M92-6a	0.41	0.09	0.13	0.06	0.15	0.74	0.08
M92-6b	0.30	0.09	0.17	0.08	0.16	0.62	0.08
M92-7a	0.29	0.09	0.20	0.07	0.15	0.71	0.08
M92-7b	0.17	0.10	0.12	0.06	0.15	0.72	0.07
M92-8a	0.26	0.09	0.15	0.07	0.14	0.72	0.08
M92-8b	0.24	0.11	0.07	0.06	0.16	0.66	0.08
M92-9a	0.29	0.09	0.23	0.07	0.13	0.60	0.06
M92-9b	0.24	0.11	0.16	0.07	0.15	0.70	0.07
M92-10a	0.28	0.09	0.12	0.07	0.15	0.82	0.07
M92-10b	0.26	0.09	0.16	0.08	0.15	0.76	0.07
M92-M1	0.43	0.12	0.26	0.08	0.20	0.22	0.10
M92-M2	0.31	0.11	0.26	0.08	0.21	0.31	0.10
M92-M3	0.22	0.10	0.22	0.08	0.19	0.30	0.09
M92-M4	0.33	0.10	0.18	0.07	0.19	0.20	0.09
M92-M5	0.25	0.09	0.19	0.07	0.17	0.47	0.08
M92-M6	0.36	0.11	0.09	0.08	0.17	0.52	0.08
M92-M7	0.30	0.08	0.11	0.05	0.18	0.80	0.08
M92-M8	0.33	0.08	0.20	0.06	0.15	0.76	0.07
M92-M9	0.32	0.08	0.23	0.07	0.14	0.67	0.06
M92-M10	0.32	0.08	0.17	0.06	0.16	0.73	0.07

[a]Uncertainty estimated from error propagation of internal counting statistical error and repeatability on BHVO2G. SE = standard error

Table S5: Concentrations and isotopic compositions measured in water samples taken from benthic chambers. The isotope analyses were conducted on the NuPlasma and NeptunePlus mass spectrometers, see Method section for details.

						NuPlasma				NeptunePlus			
Sampling time	Si	Al	Ge	Ge/Si	Al/Si*10^3	δ^{30}Si	2SD	δ^{29}Si	2SD	δ^{30}Si	2SD	δ^{29}Si	2SD
(hr)	(µM)	(µM)	(pM)	(µM M^{-1})	(µM μM^{-1})	(‰)	(‰)	(‰)	(‰)	(‰)	(‰)	(‰)	(‰)
M136/BIGO2-4; Shelf 2017; 74m water depth													
0.25	31	0.25	289	10.58	9.22					1.45	0.19	0.76	0.03
4	46												
8	70												
12	64	1.18	193	3.01	18.46	1.58	0.19	0.81	0.05	1.61	0.24	0.80	0.05
16	67												
20	71									1.41	0.09	0.69	0.03
24	75												
28	79	0.50	330	4.54	6.87	2.01	0.08	0.98	0.03	1.98	0.09	0.95	0.07

M92/BIGO1-2; Shelf 2013; 71m water depth													
0.25	54		427	7.57		1.21	0.25	0.59	0.25				
4	72												
8	81					0.89	0.12	0.41	0.19				
12	92	0.46	578	6.94	5.49	1.25	0.09	0.58	0.08	1.36	0.15	0.65	0.11
16	101												
20	109					0.91	0.17	0.50	0.08				
24	112												
28	122	0.40	262	2.41	3.72	1.15	0.16	0.45	0.09				
M137/BIGO1-5; OMZ 2017; 302 m water depth													
0.25	28	0.17	317	11.58	6.23	1.78	0.25	0.84	0.27	1.70	0.11	0.93	0.08
4	32												
8	36	0.19	234	6.87	5.55	1.44	0.18	0.62	0.15	1.64	0.10	0.89	0.02
12	40												
16	42												
20	45	0.12	372	9.03	2.81	1.58	0.14	0.84	0.15				
24	48												
28	52	0.12	206	4.72	2.80	1.52	0.24	0.69	0.18				
M136/BIGO2-2; Below OMZ 2017; 750 m water depth													
0.25	51	0.24	234	4.64	4.77	1.29	0.07	0.59	0.02				
4	52												
8	53					1.61	0.07	0.87	0.26				
12	54												
16	57												
20	57	0.13	220	4.16	2.51	1.70	0.28	0.87	0.10	1.54	0.12	0.85	0.05
24	57												
28	58	0.10	275	4.81	1.83	1.61	0.13	0.85	0.21				

3. Model set-up

3.1 Reaction-transport model for sediment pore fluids and solids

The model is based on a previous version for simulating the reactive transport of silicic acid in surface sediments (upper 50 cm) of the Peruvian shelf[4] and Guaymas Basin[5]. The turnover of solids (S) and dissolved pore fluid species (P) was simulated applying the following mass balance equations:

$$\mathrm{d_S} \cdot (1-\Phi) \cdot \frac{\partial \mathrm{S}}{\partial \mathrm{t}} = \frac{\partial}{\partial \mathrm{x}}\left(\mathrm{d_S} \cdot (1-\Phi) \cdot \left(\mathrm{D_B} \cdot \frac{\partial \mathrm{S}}{\partial \mathrm{t}} - \mathrm{w} \cdot \mathrm{S}\right)\right) + \mathrm{d_S} \cdot (1-\Phi) \cdot \mathrm{R_S} \quad (1)$$

$$\Phi \cdot \frac{\partial \mathrm{P}}{\partial \mathrm{t}} = \frac{\partial}{\partial \mathrm{x}}\left(\Phi \cdot \left(\mathrm{D_S} \cdot \frac{\partial \mathrm{P}}{\partial \mathrm{t}} - \mathrm{v} \cdot \mathrm{P}\right)\right) + \Phi \cdot \mathrm{R_S} \quad (2)$$

where S is the concentration of solid species in dry sediment (g g^{-1}), P is the concentration of dissolved species in pore water (µmol cm^{-3}), t is time (yr), x is sediment depth (cm), d_S is the density of dry solids (g cm^{-3}), Φ is sediment porosity (-), D_B is the bioturbation coefficient (cm^2 yr^{-1}); w is the burial velocity of solids (cm yr^{-1}); R_S is the turnover rates of solid species (g g^{-1} yr^{-1}), R_D is the turnover rate of dissolved species (mmol cm^{-3} yr^{-1}), D_S is the molecular diffusion coefficient of solutes in porewater (cm^2 yr^{-1}); and v is the burial velocity of pore water (cm yr^{-1}). Parameter values, depth-dependent functions, kinetic rate laws, and rate terms applied in the model are listed in Tables S6 – S9.

The model was set up for five solid species (SiO_2 in biogenic opal ($bSiO_2$), SiO_2 in authigenic phases, K in sediments, $^{30}SiO_2$ in biogenic opal, $^{30}SiO_2$ in authigenic phases) and four species dissolved in porewater (H_4SiO_4, $H_4{}^{30}SiO_4$, K, Ge).

Constant fluxes (rain rates, RR_S) were applied for the solids at the upper boundary of the model (x = 0):

$$d_S \cdot (1 - \Phi) \cdot \left(-D_B \cdot \frac{\partial S}{\partial t} + w \cdot S\right)\Big|_{x=0} = RR_S \tag{3}$$

whereas constant concentrations corresponding to ambient bottom water values (P_{BW}) were used for the solutes:

$$P|_{x=0} = P_{BW} \tag{4}$$

A zero gradient condition was applied at the lower boundary (x = L) for both solids and solutes (except dissolved K):

$$\frac{\partial S}{\partial x}\Big|_{x=L} = 0 \qquad \frac{\partial P}{\partial x}\Big|_{x=L} = 0 \tag{5}$$

The linear decrease in dissolved K concentrations with depth suggests that a non-zero gradient is more suitable for this variable:

$$\frac{\partial K}{\partial x}\Big|_{x=L} = -\frac{J_K}{\varphi \cdot D_S} \tag{6}$$

where J_K is the prescribed flux of K (3.65 µmol cm^{-2} yr^{-1}).

Fluxes, J (µmol cm^{-2} yr^{-1}), of each solute, P, at the sediment surface were calculated with the model as:

$$J_P = \varphi \cdot \left(v \cdot P - D_S \cdot \frac{\partial P}{\partial x}\right)\Big|_{x=0} \tag{7}$$

The model was solved using the solver for partial differential equations of MATHEMATICA v12 applying the Method-of-Lines approach. The modeling was done in two stages. In the first step, the model was integrated over time until a steady state was attained. The results from this simulation are representative of the situation during 2013 that was unaffected by the coastal El Niño event (Fig. S4). The steady state concentration-depth profiles from the first step then served as initial conditions for a simulation where the model was integrated for 120 days. This time frame corresponds to the period between the beginning of the coastal El Niño in January 2017[6,7] and fieldwork during M136 in April/May 2017. In this second step, rates of the dissolution of a terrigenous phase (albite, $NaAlSi_3O_8$) and authigenic clay precipitation were increased to reproduce the silicon isotope data and Ge/Si ratios in pore fluids (Fig. S4). More advanced simulations would be required to model the sudden deposition and diagenesis of a sediment layer of known thickness and bulk mineralogy onto the sediment surface.

The isotope modeling is based on previous isotope model to simulate the reactive transport of dissolved silicate in surface sediments[4]. Separate mass balance equations (see above) were set up to simulate the turnover of total dissolved $H_4{}^{30}SiO_4$ and H_4SiO_4. The isotopic composition of the pore fluid was calculated as ratio of these two compounds (MF_{30} = $^{30}Si/Si$). Considering the abundance of the three Si isotopes ^{28}Si, ^{29}Si and ^{30}Si, the mole fraction MF_{30} is related to the commonly used isotope ratio (R_{30} = $^{30}Si/^{28}Si$) as:

$$R_{30} = \frac{MF_{30} \cdot R_{St30} \cdot (R_{St29} \cdot (c_R - 1) - 1)}{c_R \cdot MF_{30} \cdot R_{St29} + R_{St30} \cdot (MF_{30} - 1)} \tag{8}$$

with R_{St30} = 0.0341465, R_{St29} = 0.0507446 and c_R = 0.51[4].

The δ^{30}Si value of the pore fluid (in ‰) is calculated from the ratio as:

$$\delta^{30}\mathrm{Si} = \left(\frac{\mathrm{R}_{30}}{\mathrm{R}_{\mathrm{St30}}} - 1\right) \cdot 1000 \quad (9)$$

The δ^{30}Si of the silicic acid flux ($J_{\delta 30}$, ‰) from the sediment to the overlying water was calculated from the fluxes of ^{30}Si (J_{30}) and the total Si flux (J_{tot}) as:

$$J_{\delta 30} = \left(\frac{\frac{J_{30}}{J_{tot}} \cdot \mathrm{R}_{\mathrm{St30}} \cdot (\mathrm{R}_{\mathrm{St29}} \cdot (c_{\mathrm{R}} - 1) - 1)}{c_{\mathrm{R}} \cdot \frac{J_{30}}{J_{tot}} \cdot \mathrm{R}_{\mathrm{St29}} + \mathrm{R}_{\mathrm{St30}} \cdot \left(\frac{J_{30}}{J_{tot}} - 1\right)}\right) \cdot \frac{1}{\mathrm{R}_{\mathrm{St30}}} - 1 \quad (10)$$

Concentrations of biogenic opal, K/Al ratios in bulk sediment and the concentration and isotopic composition of silicic acid determined in pore fluids were employed to constrain rates of opal and terrigenous phase dissolution and the precipitation rate of authigenic phases. The isotopic composition of dissolved silicate in bottom water at the shelf site ($\delta^{30}Si_{bw}$ = +1.4 ‰) is the same as previous bottom water values measured in the OMZ at 11 °S[4]. The isotopic composition of biogenic silica on the shelf was not measured. However, since pore fluid silicic acid isotopes ($\delta^{30}Si_{pf}$) measured during M92 show very little change with depth (1.2 ± 0.1 ‰), this value was used for the δ^{30}Si of biogenic silica ($\delta^{30}Si_{bSiO2}$). This assumes that authigenic Si precipitation or clay mineral dissolution rates during M92 fieldwork were too low to greatly impact the $\delta^{30}Si_{pf}$ data, although the Ge data point toward low background rates (see below). $\delta^{30}Si_{bSiO2}$ values measured in surface sediments in the OMZ at 11 °S (145 m water depth) were +1.03 ± 0.15 ‰ and agree within error with our assumed value.

During M136, authigenic clay precipitation was assumed to be the reason for the significant positive shift in the pore fluid composition. Authigenic phases precipitating in marine sediments are depleted in ^{30}Si with respect to the pore fluids. We derived a fractionation factor of Δ_{Au} = -3 ‰ ($\Delta_{Au} = \delta^{30}Si_{au} - \delta^{30}Si_{pf}$) by iteration, further consolidated by sensitivity tests (see supplementary section 3.3) to simulate the impact of authigenic phase precipitation on pore fluid composition as observed in Peruvian OMZ sediments. This is the same fractionation factor as used for simulating Si isotopes involved in authigenic clay precipitation in the Mariana Trench[8]. With this approach, the positive shift of $\delta^{30}Si_{pf}$ values observed in the upper 5 cm sediment was reproduced in the model by a combination of opal dissolution and authigenic phase precipitation. Following Ehlert et al.[4], the opal dissolution rate and authigenic mineral precipitation rate decrease exponentially with sediment depth (Table S7 and Fig S3) and a solubility control was applied to simulate both rates (Table S8).

Dissolution of albite, detected by XRD (Table S3), was assumed to be co-occurring alongside authigenic clay precipitation. The dissolution rate was also described as an exponential decrease with depth (Fig. S3). The isotopic composition of this phase subject to dissolution ($\delta^{30}Si_{terr}$) was set to -0.4 ‰[9]. The Ge/Si ratio in the terrigenous phase was fixed at 3.1×10^{-6} following Kurtz et al.[10]. Opal dissolution and authigenic clay precipitation also impact Ge concentrations. Opal dissolution releases Ge to the pore fluid whereas authigenic clay precipitation removes Ge. The Ge/Si ratios in opal were fixed at 0.76×10^{-6} µmol/mol following Sutton et al.[11]. The Ge/Si ratio in authigenic clays is more uncertain and this parameter was tuned to the Ge data. The best fit value was 1.1×10^{-6} µmol/mol, which agrees with experimental studies by Fernandez et al.[12] who showed that the rate of Ge uptake is many orders of magnitude lower than for Si. All three processes were needed to properly simulate the Si and $\delta^{30}Si_{pf}$ data in addition to Ge concentrations and Ge/Si ratios in the pore fluids and K concentrations.

K/Al ratios measured in the solid phase and pore fluid K data were used as additional model constraints. We did not separate the pore fluid from the sediment prior to the solid phase K analysis. Hence, the K concentrations measured in the dried sediment samples were corrected for the contribution of K dissolved in pore fluids applying the following equation[4]:

$$K_C = K_M - \frac{f_W}{1-f_W} K_{PW} \qquad (11)$$

where K_C is the concentration of K in the solid phase (g/g) corrected for the pore water contribution, K_M is the concentration of K measured in dried samples (g/g), f_W is the initial water content of the wet samples prior to drying (g/g) and K_{PW} is the concentration of dissolved K in the pore water (g/g). The concentration of K in pore water was measured in units of mmol/dm^3. It was converted into appropriate units (g/g) by dividing by the factor f_{sp} (Table S7) using the molecular weight of K.

The uptake of K in authigenic phases was simulated applying a K/Si ratio following Geilert et al.[5]. Authigenic Si phases formed in marine sediments are typically enriched in K and we applied the average K/Si of the Peruvian authigenic minerals (K/Si_{Au} = 0.22 mol/mol; see main text) to define the uptake of K in authigenic phases[13]. We also applied a constant Al content of 5 % in the model corresponding to the mean Al content measured in the shelf sediments (5.3 ± 0.9 wt.%). This assumes that the Al deposition rate at the sediment surface is constant, and that Al released from sediments during mineral dissolution was quantitatively re-precipitated in authigenic minerals[5]. Simulated particulate K concentrations were normalized to this value and compared to the measured K/Al ratios. With this approach, changes in the solid Al concentrations observed in the data are implicitly assumed to be caused by processes that are not considered in the model, such as changes in the composition and mass accumulation rate of terrigenous phases deposited at the sediment surface over time. A best fit model solution to the observed data set was obtained by systematically varying the kinetic rate constants (Fig. S3, Table S7).

3.2. Simulation of benthic chamber data

The modeled fluxes (Eq. 7) served as source/sink terms for the ordinary differential equations that describe concentration changes (dP/dt) in the benthic chambers. The fluxes were converted from µmol cm^{-2} yr^{-1} to µmol dm^{-3} h^{-1} by multiplying by the factor 10/365/24/hw, where hw is the height of the water in the chamber (0.2 m). The $\delta^{30}Si$ of silicic acid in the chamber was calculated using the equation above. Bottom water concentrations from the reaction transport model were used as initial conditions. The model was run over the time period of the chamber incubations (34 h). Measured and simulated H_4SiO_4 and $H_4{}^{30}SiO_4$ in benthic chambers are shown in Fig. S5.

3.3. Sensitivity tests

Further model runs were conducted to study how the isotopic composition of dissolved silica is controlled by the isotopic fractionation that occurs during the precipitation of authigenic phases. Using a similar model, Ehlert et al. (2016) derived a fractionation factor Δ_{au} of -2 ‰, which is less than our baseline case of -3 ‰. We therefore attempted to simulate our post El Niño data with Δ_{au} = -2 ‰. Although a reasonable fit to porewater $\delta^{30}Si$ using Δ_{au} = -2 ‰ can be achieved provided that the maximum rate of authigenic Si precipitation is simultaneously increased by a factor of six (Fig. S6B, solid black curve), the enhanced precipitation depletes porewater in dissolved Si (Fig. S6A, solid black curve). To compensate for this discrepancy, opal dissolution was increased five-fold to provide a source of isotopically-enriched Si, resulting again in a reasonable fit to porewater Si and $\delta^{30}Si$ (dashed black

curves). However, as a result of solubility feedbacks on authigenic precipitation rates (Table S8), opal dissolution stimulates further authigenic Si precipitation and causes a large depletion in dissolved K and increase in particulate K/Al ratios (Fig. S6C and D). These deviations are much higher than can be reasonably explained by typical seasonal variations in K and Al. Hence, we conclude that for the current model configuration the precipitation of authigenic phases is associated with a -3 ‰ fractionation factor.

Table S6. Parameter values applied in the model. "Method" refers to the procedure by which parameter values are constrained: A = Measurements, B = Literature values, C = Model Fitting. References: [1] Dale et al. (2016)[14], [2] Dale et al. (2021)[15], [3] Wollast and Garrels (1971)[16], [4] Schulz (2000)[17], [5] Michalopoulos and Aller (2004)[13], [6] Sutton et al. (2010)[18], [7] Kurtz et al. (2002)[10], [8] Savage et al. (2012)[9].

Parameter	Symbol	Value	Method
Sediment temperature [a]	T	15 °C	A [1]
Salinity of porewater	Sal	35	A [1]
Porosity at x = 0	Φ_0	0.94	A [1]
Porosity at x = infinity	Φ_f	0.87	A [1]
Attenuation coefficient for porosity decrease	px	0.15 cm^{-1}	A [1]
Burial velocity after compaction	w_f	0.47 cm yr^{-1}	A [2]
Density of dry solids	d_S	2.5 g cm^{-3}	A [1]
Bioturbation coefficient at x = 0	$D_B(0)$	13 cm^2 yr^{-1}	A [2]
Depth of bioturbated zone	x_B	0.5 cm	A [2]
Molecular weight of SiO_2	MW_{SiO2}	60.08 g mol^{-1}	
Molecular weight of K	MW_K	39.098 g mol^{-1}	
Molecular diffusion coefficient for H_4SiO_4	D_M	241 cm^2 yr^{-1}	B [3]
Molecular diffusion coefficient for K	D_M	494 cm^2 yr^{-1}	B [4]
Molecular diffusion coefficient for Ge [b]	D_M	241 cm^2 yr^{-1}	C [This work]
Solubility of biogenic opal [c]	Sol_{opal}	0.9 mM	C [This work]
Solubility of authigenic phase	Sol_{au}	0.2 mM	C [This work]
Bottom water H_4SiO_4 concentration	$H_4SiO_{4\_BW}$	0.039 mM	A [This work]
Bottom water $H_4{}^{30}SiO_4$ concentration [d]	$H_4{}^{30}SiO_{4\_BW}$	0.0123 mM	A [This work]
$\delta^{30}Si$ of H_4SiO_4 in bottom water	$\delta^{30}Si_{BW}$	1.4 ‰	A [This work]
Bottom water K concentration	K_{BW}	10.2 mM	A [This work]
Bottom water Ge concentration	Ge_{BW}	$200 \cdot 10^{-9}$	A [This work]
Rain rate of biogenic opal[e]	RR_{opal}	36.7 mg cm^{-2} yr^{-1}	A [This work]
Rain rate of K	RR_K	2.4 mg cm^{-2} yr^{-1}	A [This work]
Molar K/Si ratio in authigenic phase	K/Si_{au}	0.22	B [5]
Molar Ge/Si ratio in biogenic opal	Ge/Si_{opal}	$0.76 \cdot 10^{-6}$	B [6]
Molar Ge/Si ratio in authigenic phase	Ge/Si_{au}	$1.1 \cdot 10^{-6}$	C [This work]
Molar Ge/Si ratio in terrigenous phase	Ge/Si_{terr}	$3.1 \cdot 10^{-6}$	B [7]
$\delta^{30}Si$ of opal deposited at the seafloor	$\delta^{30}Si_{opal}$	+1.2 ‰	A [This work]
$\delta^{30}Si$ of dissolving terrigenous phases	$\delta^{30}Si_{terr}$	-0.4 ‰	B [8]
Fractionation factor for authigenic phase formation	Δ_{au}	-3.0 ‰	C [This work]
Fractionation factor for authigenic phase formation in term of mol fractions[f]	β_{au}	0.99717	C [This work]

[a] The mean bottom water temperature on the shelf during M92 and M136 was 14 °C and 15.8 °C, respectively. A value of 15 °C was used for both simulations.

[b] Assumed to be equivalent to dissolved silicic acid[19].

[c] The theoretical opal solubility was 1177 µM during M92 and 1236 µM during M136; the difference due to the higher bottom water temperature during M136. The lower value used in the model was

based on the observed asymptotic silicic acid concentrations and attributed to chemical interactions between silica shell fragments and dissolved Al released from detrital feldspar minerals[20–22].

[d] $H_4{}^{30}SiO_4$ in the bottom water is calculated as $\frac{H_4SiO_{4\_BW} \cdot R_{30\_BW} \cdot R_{St30}}{c_R \cdot R_{St29} \cdot (R_{30_{BW}} - R_{St30}) + R_{St30} \cdot (1 + R_{30_{BW}} + R_{St29})}$, where the $^{30}Si/^{28}Si$ of the bottom water (R_{30_BW}) is $\left(\frac{\delta^{30}Si_{BW}}{1000} + 1\right) \cdot R_{30}$

[e] Fit to the SiO_2 data

[f] $\beta_{au} = \alpha P^{1/1.058}$, where $\alpha P = \exp(\Delta_{au}/1000)$

Table S7. Depth-dependent functions applied in the model.

Parameter	Symbol	M92	M136
Porosity	Φ	$\Phi_f + (\Phi_0 - \Phi_f) \cdot \exp^{(-px \cdot x)}$	same as M92
Bioturbation coefficient	D_B	$D_B(0) \cdot \exp\left(-\frac{x^2}{2 \cdot x_B^2}\right)$	same as M92
Burial velocity of solids	w	$w_f \cdot \frac{1-\Phi_f}{1-\Phi}$	same as M92
Burial velocity of solutes	v	$w_f \cdot \frac{\Phi_f}{\Phi}$	same as M92
Tortuosity	To^2	$1 - 2 \cdot \ln(\Phi)$	same as M92
Diffusion coefficient of solutes in porewater	D_S	$\frac{D_M}{To^2} + D_B$	same as M92
Function to convert rates from g g^{-1} yr^{-1} to µmol cm^{-3} yr^{-1}	f_{sp}	$d_S \cdot 10^6 \cdot \frac{1-\Phi}{\Phi \cdot MW}$	same as M92
Kinetic constant for biogenic opal dissolution (in yr^{-1})	k_{opal}	$1 \cdot e^{-x/3}$	same as M92
Kinetic constant for authigenic mineral precipitation (in µmol cm^{-3} yr^{-1})	k_{au}	$12 \cdot e^{-x/0.8}$	$400 \cdot e^{-x/0.8}$
Kinetic constant for dissolution of terrigenous phase (in g SiO_2 $g^1 yr^{-1}$)	k_{terr}	$0.0035 \cdot e^{-x/1.8}$	$0.025 \cdot e^{-x/1.8}$
Mole fraction of Si	MF	$\frac{^{30}Si}{Si}$	same as M92

Table S8: Kinetic rate laws applied in the model.

Reaction	Symbol	Equation
Biogenic opal dissolution in g g^{-1} yr^{-1}	R_{opal}	$k_{opal} \cdot bSiO_2 \cdot \left(1 - \frac{H_4SiO_4}{Si_{opal}}\right)$
Dissolution of $^{30}SiO_2$ of terrigenous phase in g g^{-1} yr^{-1}	R_{terr}	k_{terr}
Precipitation of authigenic Si in µmol cm^{-3} yr^{-1}	R_{au}	$k_{au} \cdot \left(\frac{H_4SiO_4}{Si_{au}} - 1\right)$
Dissolution of $^{30}SiO_2$ in biogenic opal in g g^{-1} yr^{-1}	R_{opal}^{30}	$k_{opal} \cdot {}^{30}bSiO_2 \cdot \left(1 - \frac{H_4SiO_4}{Si_{opal}}\right)$
Dissolution of $^{30}SiO_2$ of terrigenous phase in g g^{-1} yr^{-1}	R_{terr}^{30}	$MF_{terr} \cdot R_{terr}$
Precipitation of authigenic ^{30}Si in µmol cm^{-3} yr^{-1}	R_{au}^{30}	$MF_{H_4SiO_4} \cdot \beta_{au} \cdot R_{au}$

Table S9: Rate terms applied in mass balance equations.

Species	Equation
Biogenic opal (in g g^{-1} yr^{-1})	- R_{opal}
^{30}Si in biogenic opal (in g g^{-1} yr^{-1})	$-R_{opal}^{30}$
Authigenic Si phase (in g g^{-1} yr^{-1})	+ R_{au} / f_{sp}
^{30}Si in authigenic Al phase (in g g^{-1} yr^{-1})	+ R_{au}^{30} / f_{sp}
K in sediment (in g g^{-1} yr^{-1})	$K/Si_{au} \cdot R_{au} / f_{sp}$
Dissolved silica (in µmol cm^{-3} yr^{-1})	$f_{sp} \cdot R_{opal} + R_{terr} – R_{au}$
^{30}Si in dissolved silica (in µmol cm^{-3} yr^{-1})	$f_{sp} \cdot R_{opal}^{30} + R_{terr}^{30} – R_{au}^{30}$
Dissolved K (in µmol cm^{-3} yr^{-1})	$- K/Si_{au} \cdot R_{au}$
Dissolved Ge (in µmol cm^{-3} yr^{-1})	$f_{sp} \cdot R_{opal} \cdot Ge/Si_{opal} – R_{au} \cdot Ge/Si_{au} + R_{terr} \cdot Ge/Si_{terr}$

Table S10: Model results obtained at the end of the simulations (M92 = steady state; M136 = non-steady state).

Process	M92	M136
SiO_2 (opal) dissolution, µmol Si cm^{-2} yr^{-1}	247	367
Ge release from opal dissolution, p mol Ge cm^{-2} yr^{-1}	188	279
Authigenic Si mineral precipitation, µmol Si cm^{-2} yr^{-1}	13	291
Authigenic Ge mineral precipitation, pmol Ge cm^{-2} yr^{-1}	14	320
Authigenic K mineral precipitation, µmol K cm^{-2} yr^{-1}	2.9	64
Dissolution of terrigenous phase, µmol Si cm^{-2} yr^{-1}	20	140
Dissolution of terrigenous phase, pmol Ge cm^{-2} yr^{-1}	61	435
Benthic efflux of silicic acid, µmol Si cm^{-2} yr^{-1} [a]	253	215
δ^{30}Si of silicic acid flux, ‰ [b]	+1.25	+2.1
Mean δ^{30}Si of fresh authigenic Si post El Niño, ‰ [c]	-	-0.56

[a] Measured silicic acid fluxes at St. 533BIGO2-4 during M136 range from 189 to 281 µmol Si cm^{-2} yr^{-1}.

[b] Calculated using Eq. (10).

[c] Mean value for the sediment layer between the sediment surface and the depth where Si_{au} falls to 1 % of its surface value.

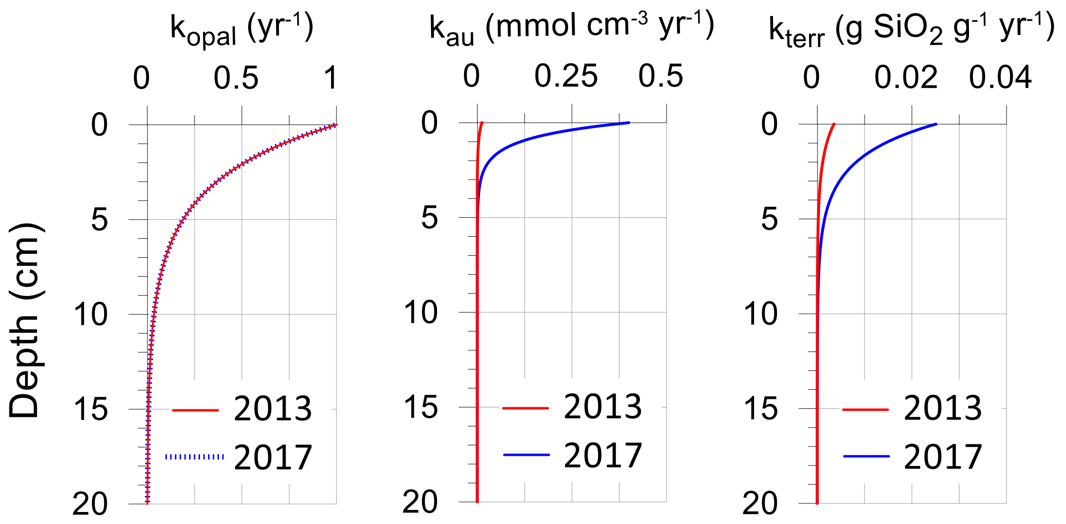

Fig. S3. Down-core profiles of kinetic constants for biogenic opal dissolution (k_{opal}), authigenic phase precipitation (k_{au}), and terrigenous phase dissolution (k_{terr}) applied in the model.

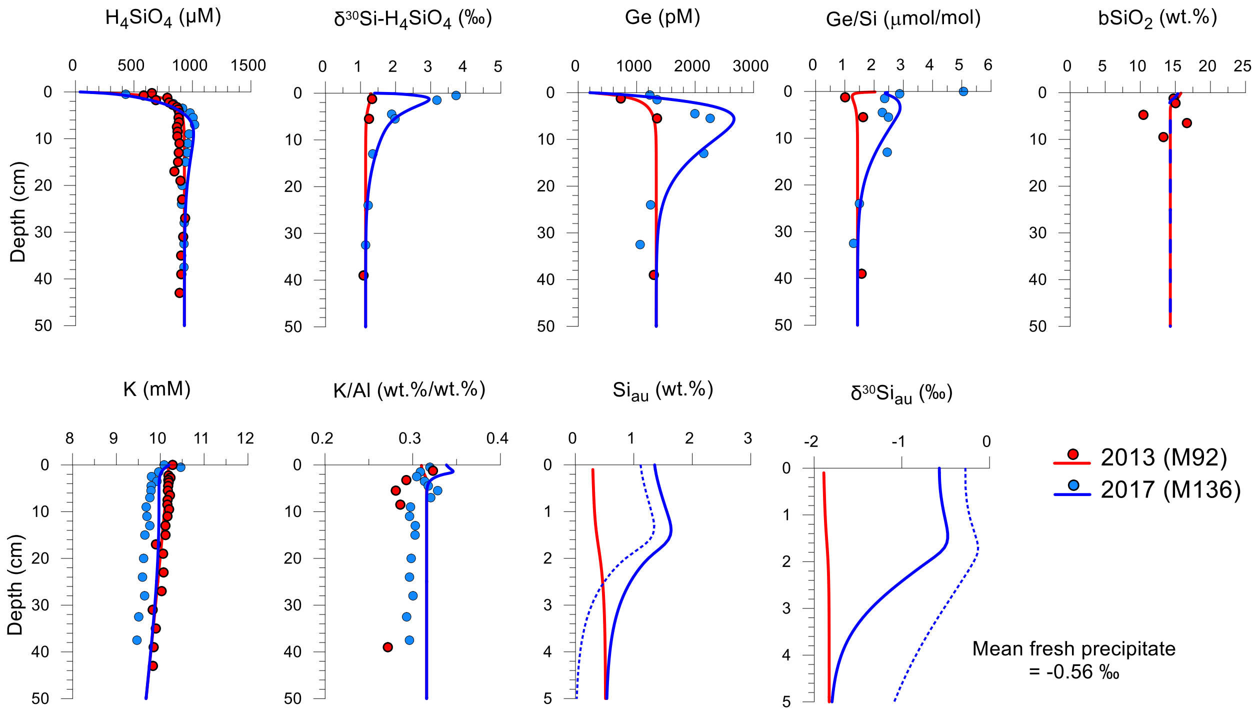

Fig. S4. Model profiles (curves) and measured (symbols) from the steady state simulation (M92, 2013, red solid curves and symbols) and the dynamic post El Niño simulation (M136, 2017, blue solid curves and symbols). The dotted blue curves of the authigenic phase correspond to the mineral freshly precipitated during the dynamic simulation (see Section 3.3). The solid blue curves in the authigenic Si plots correspond to the total phase that includes the precipitate from the steady state run and the fresh precipitate.

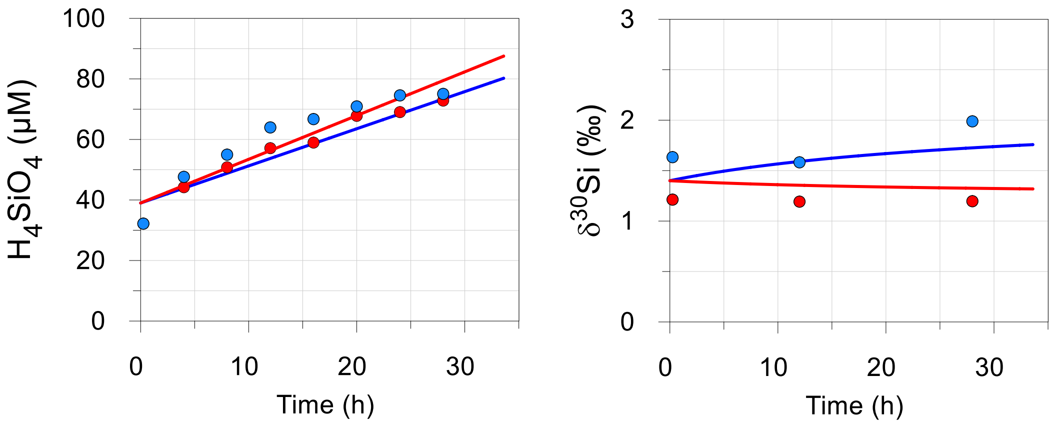

Fig. S5. Model simulation results (curves) and measurements (symbols) of silicic acid concentrations, and $\delta^{30}Si$ values in the benthic chamber incubation experiments on the shelf during 2013 (red) and 2017 (blue) post El Niño. Silicic acid concentrations during 2017 (St. 533BIGO2-4) are mean values of two benthic chambers. Silicic acid concentrations measured in 2013 (St. 110BIGO1-2) are normalized to the initial concentration from 2017.

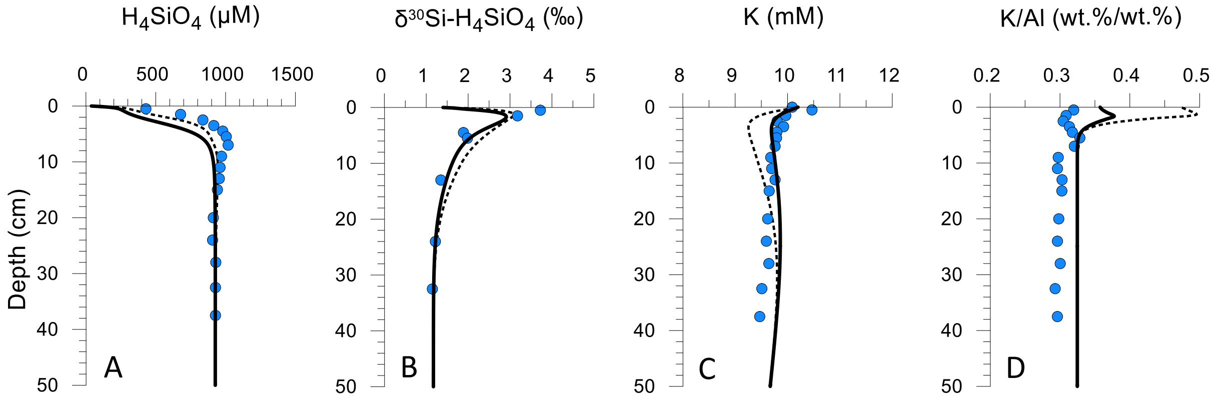

Fig. S6. Model sensitivity test results. Model results are shown for (A) silicic acid (H_4SiO_4) concentrations, (B) $\delta^{30}Si$ values, (C) K concentrations and (D) K/Al ratios. Symbols are measured data from the post El Niño simulation (M136, 2017). Results differ from the baseline dynamic model runs (Fig. S4) as follows. Solid black curves show model results with Δ_{au} = -2 ‰ and $k_{au} = 2400 \cdot e^{-x/0.8}$. Dashed black curves shows results with an additional increase of k_{opal} to $5 \cdot e^{-x/3}$.

4. Supplementary discussion

4.1 Mixing calculations

Simultaneously occurring early diagenetic sedimentary reactions can be identified using endmember mixing calculations following Grasse et al.[1] (Eq. 12). Next to the diatom and bottom water endmembers, a lower Si isotope source is required to explain the shift to low pore fluid $\delta^{30}Si$ (main text Fig. 2). Primary minerals were enriched in the sediments from the Peruvian margin (Table S3). These minerals were found to dissolve in hemipelagic sediments[23] and have lower $\delta^{30}Si$ values[24] compared to diatoms and bottom water (bw). Assuming that feldspar is the primary mineral, the following mixing calculation can be applied:

$$Sample_{mix} = source_A * f + \ source_B * (1 - f) \quad (12)$$

Where $Sample_{mix}$ is either $\delta^{30}Si_{mix}$ or Ge/Si_{mix} and $source_A$ is the $\delta^{30}Si_{bSiO2}$ *or* Ge/Si_{bSiO2} of dissolving diatoms (+1.2‰ (M92, see section S3.1); 0.76x10^{-6}) or seawater (+1.41 to 1.65‰[1,25]; 0.76x10^{-6} reference[11]) and $source_B$ is the $\delta^{30}Si_{fsp}$ *or* Ge/Si_{fsp} of feldspar (-0.4‰[9]; 3.1x10^{-6} reference[10]). Mixing fractions are represented by f, varied over 100 % diatom/ seawater and 0% affected by feldspar dissolution and vice versa.

4.2 Benthic chamber incubation experiments

The Si concentrations increased over time in the incubation experiments, showing the highest increase at the shelf stations (from 31 to 79 µM for M136 (2017) and 54 to 122 µM for M92 (2013); Fig. S2, Table S5). Si concentrations in the OMZ increased from 28 to 52 µM and from 51 to 58 µM below the OMZ. Mean Si fluxes[15] were 12.5 mmol m^{-2} d^{-1} at the shelf station during M92, 6.5 mmol m^{-2} d^{-1} at the shelf station during M136, 1.8 mmol m^{-2} d^{-1} in the OMZ, and 1.5 mmol m^{-2} d^{-1} below the OMZ. Germanium concentrations were highly variable and showed no systematic increase over time at all stations. The concentrations ranged from 193 pM to 578 pM. Accordingly, Ge/Si ratios showed no systematic trend over time and ranged from 2.4 to 11.6. Aluminium concentrations decreased over time from a maximum of 0.46 µM (shelf station 2013) to a minimum of 0.10 µM (below OMZ). Only the shelf station from 2017 showed a slight increase from 0.25 to 0.50 µM, though with a large excursion to 1.18 µM at intermediate times. The $\delta^{30}Si_{flux}$ values on the shelf (2013) and OMZ were uniform over time, on average +1.11±0.11 ‰ and +1.58±0.14 ‰, respectively (± 1SD). Below the OMZ and on the shelf in 2017, $\delta^{30}Si_{flux}$ slightly increased from +1.29 ‰ to +1.61 ‰ and from +1.45 ‰ to +1.98 ‰, respectively.

The benthic Si and Ge fluxes differed significantly and showed a decoupled behaviour. While benthic Si concentrations increased steadily, Ge concentrations were highly variable and did not follow a systematic trend (Fig. S2). Therefore, Ge/Si ratios also varied significantly with a slight trend to lower ratios over time, attributable to increasing Si concentrations. We infer that Ge was likely affected stronger by changing redox conditions compared to Si in the sediments during the incubation experiments or during chamber retrieval. Baronas et al.[26] reported a similar effect of spatial heterogeneity of Ge/Si ratios during core incubation experiments.

Benthic Fe fluxes of -22.7 mmol m^{-2} yr^{-1} were measured in 2013[27]. In contrast, during the sampling campaign in 2017, the benthic Fe flux was much lower -2.6 mmol m^{-2} yr^{-1} [27]. Plass et al.[27] attributed this lower flux to the precipitation of FeS during the oxic conditions caused by the El Niño event, which forced the disappearance of sulfur-oxidizing bacteria and the accumulation of H_2S in the surface sediments. Due to the high variability of Al and Ge over experimental time in the benthic chambers, no reliable benthic fluxes could be calculated and the benthic contribution of Al, Ge, Al/Si and Ge/Si to Peruvian bottom waters cannot be assessed.

A significant offset between bottom water and the benthic δ^{30}Si flux ($\Delta^{30}Si_{fluxfinal-bw}$) was noted for the shelf station from 2017 and the station below the OMZ with $\Delta^{30}Si_{fluxfinal-bw}$ of +0.72 ‰ and +0.63 ‰, respectively (Fig. S2). There was no obvious correlation with the benthic Si flux, since the highest Si flux (shelf 2013) and lowest Si flux (OMZ) both displayed $\Delta^{30}Si_{fluxfinal-bw}$ of 0, within analytical precision. At the 2017 shelf station, the high pore fluid δ^{30}Si values (up to +3.72 ‰; main text Fig. 2) caused by extensive authigenic clay formation (see main text) were also reflected in the δ^{30}Si values of the benthic flux (+2.1 ‰; Table S10).

Supplementary References

1. Grasse, P., Ryabenko, E., Ehlert, C., Altabet, M. A. & Frank, M. Silicon and nitrogen cycling in the upwelling area off Peru: A dual isotope approach. *Limnol. Oceanogr.* **61**, 1661–1676 (2016).
2. Krahmann, G. *et al.* Climate-biogeochemistry interactions in the tropical ocean: data collection and legacy. *Front. Mar. Sci.* **8**, (2021).
3. Jochum, K. P. *et al.* GeoReM: A New Geochemical Database for Reference Materials and Isotopic Standards. *Geostand. Geoanalytical Res.* **29**, 333–338 (2005).
4. Ehlert, C. *et al.* Stable silicon isotope signatures of marine pore waters – Biogenic opal dissolution versus authigenic clay mineral formation. *Geochim. Cosmochim. Acta* **191**, 102–117 (2016).
5. Geilert, S. *et al.* Impact of ambient conditions on the Si isotope fractionation in marine pore fluids during early diagenesis. *Biogeosciences* **17**, 1745–1763 (2020).
6. Echevin, V. *et al.* Forcings and Evolution of the 2017 Coastal El Niño Off Northern Peru and Ecuador. *Front. Mar. Sci.* **5**, 1–16 (2018).
7. Son, R. *et al.* Climate diagnostics of the extreme floods in Peru during early 2017. *Clim. Dyn.* **54**, 935–945 (2020).
8. Geilert, S., Grasse, P., Wallmann, K., Liebetrau, V. & Menzies, C. D. Serpentine alteration as source of high dissolved silicon and elevated δ^{30}Si values to the marine Si cycle. *Nat. Commun.* **11**, (2020).
9. Savage, P. S. *et al.* The silicon isotope composition of granites. *Geochim. Cosmochim. Acta* **92**, 184–202 (2012).
10. Kurtz, A. C., Derry, L. A. & Chadwick, O. A. Germanium – silicon fractionation in the weathering environment. *Geochim. Cosmochim. Acta* **66**, 1525–1537 (2002).
11. Sutton, J., Ellwood, M. J., Maher, W. A. & Croot, P. L. Oceanic distribution of inorganic germanium relative to silicon : Germanium discrimination by diatoms. *Global Biogeochem. Cycles* **24**, 1–13 (2010).
12. Fernandez, N. M., Perez-Fodich, A., Derry, L. A. & Druhan, J. L. A first look at Ge/Si partitioning during amorphous silica precipitation: Implications for Ge/Si as a tracer of fluid-silicate interactions. *Geochim. Cosmochim. Acta* **297**, 158–178 (2021).
13. Michalopoulos, P. & Aller, R. C. Early diagenesis of biogenic silica in the Amazon delta: Alteration, authigenic clay formation, and storage. *Geochim. Cosmochim. Acta* **68**, 1061–1085 (2004).
14. Dale, A. W., Sommer, S., Lomnitz, U., Bourbonnais, A. & Wallmann, K. Biological nitrate transport in sediments on the Peruvian margin mitigates benthic sulfide emissions and drives pelagic N loss during stagnation events. *Deep. Res. Part I Oceanogr. Res. Pap.* **112**, 123–136 (2016).
15. Dale, A. W. *et al.* Recycling and Burial of Biogenic Silica in an Open Margin Oxygen Minimum Zone. *Global Biogeochem. Cycles* **35**, (2021).
16. Wollast, R. & Garrels, R. Diffusion Coefficient of Silica in Seawater. *Nat. Phys. Sci.* **229**, (1971).
17. Schulz, H. D. (2000) Conceptual models and computer models. In H. D. Schulz, M. Zabel (eds) *Marine Geochemistry*, Springer-Verlag, Berlin, pp 417-442.

18. Tréguer, P. J. *et al.* Reviews and syntheses: The biogeochemical cycle of silicon in the modern ocean. *Biogeosciences* **18**, 1269–1289 (2021).

19. King, S. L., Froelich, P. N. & Jahnke, R. A. Early diagenesis of germanium in sediments of the Antarctic South Atlantic: In search of the missing Ge sink. *Geochim. Cosmochim. Acta* **64**, 1375–1390 (2000).

20. Dixit, S., Van Cappellen, P. & van Bennekom, a J. Processes controllong solubility of biogenic silica and pore water build-up of silicic acid in marine sediments. *Mar. Chem.* **73**, 333–352 (2001).

21. Dixit, S. & Van Cappellen, P. Predicting benthic fluxes of silicic acid from deep-sea sediments. *J. Geophys. Res.* **108**, 3334 (2003).

22. Van Beusekom, J. E. E., Van Bennekom, A. J., Tréguer, P. & Morvan, J. Aluminium and silicic acid in water and sediments of the Enderby and Crozet Basins. *Deep. Res. Part II Top. Stud. Oceanogr.* **44**, 987–1003 (1997).

23. Rabouille, C., Gaillard, J. F., Tréguer, P. & Vincendeau, M. A. Biogenic silica recycling in surficial sediments across the Polar Front of the Southern Ocean (Indian Sector). *Deep. Res. Part II Top. Stud. Oceanogr.* **44**, 1151–1176 (1997).

24. Savage, P. S., Georg, R. B., Williams, H. M. & Halliday, A. N. The silicon isotope composition of the upper continental crust. *Geochim. Cosmochim. Acta* **109**, 384–399 (2013).

25. Grasse, P. *et al.* Controls on the Silicon Isotope Composition of Diatoms in the Peruvian Upwelling. *Front. Mar. Sci.* **8**, (2021).

26. Baronas, J. J., Hammond, D. E., Rouxel, O. J. & Monteverde, D. R. First Look at Dissolved Ge Isotopes in Marine Sediments. *Front. Earth Sci.* **7**, (2019).

27. Plass, A. *et al.* The control of hydrogen sulfide on benthic iron and cadmium fluxes in the oxygen minimum zone off Peru. *Biogeosciences* **17**, 3685–3704 (2020).
